# Molecular identification of sulfadoxine-pyrimethamine resistance in malaria infected women who received intermittent preventive treatment in the Democratic Republic of Congo

**DOI:** 10.1186/s12936-017-2160-x

**Published:** 2018-01-09

**Authors:** Emrah Ruh, Jean Paul Bateko, Turgut Imir, Aysegul Taylan-Ozkan

**Affiliations:** 1 Department of Medical Microbiology and Clinical Microbiology, Near East University Faculty of Medicine, Near East Boulevard, 99138 Nicosia, Northern Cyprus; 2 Department of Medical Biology, Higher Institute of Medical Technology, Bandundu, Democratic Republic of the Congo; 3 Department of Medical Microbiology, Hitit University Faculty of Medicine, Corum, Turkey

**Keywords:** *Plasmodium falciparum*, Sulfadoxine-pyrimethamine, Drug resistance, *Pfdhfr*, *Pfdhps*, Democratic Republic of Congo

## Abstract

**Background:**

Point mutations in *Plasmodium falciparum* dihydrofolate reductase *(Pfdhfr)* and dihydropteroate synthase *(Pfdhps)* genes which confer resistance to sulfadoxine-pyrimethamine (SP) occur at increasing rates. The present study aimed to identify *Pfdhfr* and *Pfdhps* mutations in *P. falciparum* isolates recovered from women who received two doses of SP during pregnancy in Bandundu, the Democratic Republic of Congo (DRC).

**Methods:**

A total of 48 women with confirmed *P. falciparum* infection were enrolled in the study. Finger-prick blood samples that were collected on filter paper at the time of delivery were used for DNA isolation. *Pfdhfr* and *Pfdhps* genes were amplified by a nested PCR protocol. DNA sequencing was performed on both strands, and the point mutations were analysed.

**Results:**

All of the 48 (100.0%) *P. falciparum* isolates carried at least one polymorphism in both genes. The wild-type haplotypes of *Pfdhfr* (CNCSI [C50, N51, C59, S108, I164]) and *Pfdhps* (SAKAA [S436, A437, K540, A581, A613]) were not observed in the study. In *Pfdhfr*, N51I (85.4%), C59R (60.4%), and S108N (100.0%) polymorphisms were detected. Triple mutation (CIRNI) (mutant amino acids are underlined) was the most prevalent (47.9%) *Pfdhfr* haplotype. In the study, all *P. falciparum* isolates (100.0%) harboured the A437G allele in *Pfdhps* gene. Also, K540E and A581G polymorphisms were observed in one (2.1%) isolate. Single mutant haplotype (SGKAA) was detected in 97.9% of the isolates. Mutant *Pfdhfr* and *Pfdhps* allele combinations revealed quintuple (CICNI-SGEGA; 2.1%), quadruple (CIRNI-SGKAA; 47.9%), triple (CICNI-SGKAA; 35.4%, CNRNI-SGKAA; 12.5%), and double (CNCNI-SGKAA; 2.1%) haplotypes.

**Conclusions:**

In the study, the rate of SGEGA haplotype was low (2.1%). Although K540E and A581G alleles are more common in Eastern Africa, a distinct lineage of SGEGA is also present in the DRC, which is located in Central Africa. This haplotype is associated with decreased efficacy of SP in pregnant women and infants, therefore, it should be carefully considered in the DRC and SP resistance should be routinely monitored.

## Background

The second highest rate of global malaria prevalence is documented in the Democratic Republic of Congo (DRC) [1]. Affecting a number of risk groups, malaria is also a major concern in pregnancy. More than half of the pregnant women at risk of malaria infection live in sub-Saharan Africa [2]. The infection leads to maternal anaemia and low birth weight (LBW), which results in infant mortality [2]. For this reason, the World Health Organization (WHO) recommends the administration of intermittent preventive treatment in pregnancy with sulfadoxine-pyrimethamine (IPTp-SP) [3]. The WHO guidelines that were updated in 2013 ensure a minimum of three doses of SP in pregnancy [3].

In the DRC, approximately 97% of the population lives in areas with stable malaria transmission that lasts for 8–12 months of the year [4]. In the country, IPTp was adopted in 2003 and SP has been used to prevent malaria in pregnant women and the newborns. Nevertheless, IPTp coverage has been reported to be low. The percentage of women receiving at least two doses of SP only increased from 5 to 14% over 2007–2013 period [4]. IPTp guidelines in the DRC were also revised in 2013 according to the WHO recommendations. The current target is administration of two and three doses of IPTp to a minimum of 60 and 30% of pregnant women, respectively [4].

Increasing resistance is of concern because this can limit the antimalarial activity of SP [5]. Previously in the DRC, SP treatment failure by day 28 was reported to be between 2 and 60%. Thus, SP was replaced by artemisinin-based combination therapy (ACT) as the first-line treatment of malaria [2]. Point mutations in *Plasmodium falciparum* dihydrofolate reductase *(Pfdhfr)* and dihydropteroate synthase *(Pfdhps)* genes, encoding the related DHFR and DHPS enzymes in the folate-pathway, cause resistance against pyrimethamine and sulfadoxine, respectively [6]. Notably, SP resistance is initiated by single polymorphisms, and augmented by accumulation of mutations in *Pfdhfr* and *Pfdhps* genes [7]. Five mutations have been widely reported in Africa, which are N51I, C59R, S108N in *Pfdhr*; with A437G and K540E in *Pfdhps* gene [8]. In addition to K540E, A581G polymorphism in *Pfdhps* has been typically detected in East Africa [5]. Mutant *Pfdhfr*–*Pfdhps* haplotypes lead to high level resistance which is associated with decreased efficiency of IPTp-SP [5]. Moreover, the emergence of a sextuple mutant genotype, characterized with triple *Pfdhfr* (N51I + C59R + S108N) and triple *Pfdhps* (A437G + K540E + A581G) mutations, has been associated with reduced birth weight [9].

So far, a number of reports have shown the gene polymorphisms associated with SP resistance in the DRC [10–13]. However the molecular basis of resistance among women receiving IPTp-SP in the country remains unclear. Thus, the present study aimed to identify the point mutations in *Pfdhfr* and *Pfdhps* genes in *P. falciparum* isolates recovered from newly delivered women who received two doses of SP prophylaxis during pregnancy in the DRC.

## Methods

### Study area, study population and collection of blood samples

**Fig. 1:**
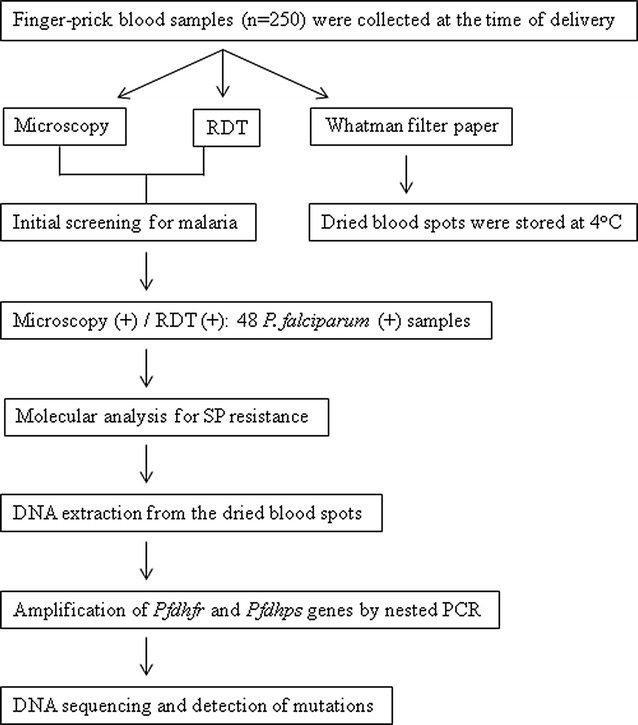
Flow chart of the study protocol. (*RDT* rapid diagnostic test)

The DRC is one of the sub-Saharan African countries and located in the central region of the continent. This study was conducted in Bandundu city, which is situated in the west of the DRC. Between March and May (rainy season), and in September (end of the dry season) in 2014, a total of 250 consecutive women who gave birth at three different health centres in Bandundu were screened for malaria infection. All of the individuals received two doses of IPTp-SP at the 16th and 28th weeks of gestation. At the time of delivery, finger-prick blood samples were collected to perform microscopy and rapid diagnostic test (RDT). Additionally, blood samples were spotted and dried on Whatman filter paper. The presence of malaria infection was determined by microscopic examination of blood smears and evaluation of RDT results (SD Bioline Malaria Ag P.f./Pan). Of the 250 women, 48 were found positive for *P. falciparum* by both microscopy and RDT. Eventually, the 48 women [mean and median age: 27.6 ± 6.5 and 27.5 (16.0–38.0), respectively] with confirmed malaria infection were included in the study and their dried blood spots were used for further molecular analysis. Flow chart of the study protocol is given in Fig. 1.

### Ethical approval

The ethical approval for the study was obtained from the Ankara Numune Training and Research Hospital Ethics Committee (Project no: E-14-286). Informed consent was collected from all participants.

### Molecular analysis of the resistance genes

Molecular tests were performed by using the dried filter paper blood spots which were collected at the time of delivery and stored in sealed plastic bags at 4 °C (Fig. 1). DNA isolation from the dried blood spots was conducted according to the manufacturer’s protocol (EURx GeneMATRIX Bio-Trace DNA Purification Kit). For amplification of *Pfdhfr* and *Pfdhps* genes, nested PCR assay was performed as described elsewhere [7]. Briefly, *Pfdhfr* gene was amplified by using the primers AMP-1 F and AMP-2R, which was followed by the nested reaction with primers M1 and M5. The 648-base pair fragment of the second PCR product covered the codons, which are associated with pyrimethamine resistance. For *Pfdhps* gene, following the first round PCR with primers M3717F and 186R, the nested reaction was performed by using the primers Rc and Rd. The 728-bp fragment of the second amplicon covered the codons, which are related with resistance to sulfadoxine. Finally, the purified PCR products were sequenced in both strands and the single nucleotide polymorphisms (SNPs) were analysed [7]. The sequences of *Pfdhfr* and *Pfdhps* genes were submitted to GenBank under the accession numbers MG254624–MG254671 and MG254672–MG254719, respectively.

## Results

### *Pfdhfr* gene polymorphisms

Of the 48 *P. falciparum* isolates sequenced, N51I (n = 41, 85.4%), C59R (n = 29, 60.4%), and S108N (n = 48, 100.0%) polymorphisms were detected. No mutation was observed at the codons 16, 50 and 164 (Table 1). The wild-type *Pfdhfr* haplotype (CNCSI [C50, N51, C59, S108, and I164]) was not identified in any of the isolates. Twenty-three (47.9%) of 48 isolates were triple mutants (CIRNI) (SNPs are underlined). Number of double mutant haplotypes (CICNI, and CNRNI) was 18 (37.5%) and six (12.5%), respectively; while one (2.1%) isolate was single mutant (CNCNI) (Table 2).

**Table 1:** Types of SNPs detected in *Pfdhfr* and *Pfdhps* genes among 48 *Plasmodium falciparum* isolates

Mutant alleles in *Pfdhfr* gene^a^	Number of the isolates, n (%)	Mutant alleles in *Pfdhps* gene^b^	Number of the isolates, n (%)
N51I	41/48 (85.4)	A437G	48/48 (100.0)
C59R	29/48 (60.4)	K540E	1/48 (2.1)
S108N	48/48 (100.0)	A581G	1/48 (2.1)

^a^*Pfdhfr* polymorphisms: codon 51, asparagine to isoleucine; codon 59, cysteine to arginine; codon 108, serine to asparagine

^b^*Pfdhps* polymorphisms: codon 437, alanine to glycine; codon 540, lysine to glutamic acid; codon 581, alanine to glycine

**Table 2:** Types of mutant *Pfdhfr* and *Pfdhps* haplotypes among 48 *Plasmodium falciparum* isolates

Haplotype	*Pfdhfr* gene^a^	Number of the isolates, n (%)	*Pfdhps* gene^b^	Number of the isolates, n (%)
Wild-type	CNCSI	0/48 (0.0)	SAKAA	0/48 (0.0)
Triple	CIRNI	23/48 (47.9)	SGEGA	1/48 (2.1)
Double	CICNI	18/48 (37.5)	–	–
Double	CNRNI	6/48 (12.5)	–	–
Single	CNCNI	1/48 (2.1)	SGKAA	47/48 (97.9)

^a^*Pfdhfr* wild-type haplotype (CNCSI [C50/N51/C59/S108/I164]); triple (51I/59R/108N); double (51I/108N, or 59R/108N); and single (108N) mutant

^b^Wild-type *Pfdhps* haplotype (SAKAA [S436/A437/K540/A581/A613]); triple (437G/540E/581G); and single (437G) mutant

### *Pfdhps* gene polymorphisms

All *P. falciparum* isolates (n = 48, 100.0%) had A437G mutation. K540E and A581G polymorphisms were observed in one (2.1%) isolate. No mutation was found at the codons 436 and 613 (Table 1). The wild-type *Pfdhps* haplotype (SAKAA [S436, A437, K540, A581, and A613]) was not detected in the study. In total, 47 (97.9%) of the isolates were single mutant (SGKAA), and one (2.1%) isolate had triple mutant haplotype (SGEGA) (Table 2).

### Combination of *Pfdhfr* and *Pfdhps* haplotypes

In the study, all of the *P. falciparum* isolates (n = 48, 100.0%) carried at least one polymorphism in both genes. Mutant *Pfdhfr* and *Pfdhps* alleles detected in the 48 isolates were combined and five different *Pfdhfr*–*Pfdhps* haplotypes were generated. Accordingly, one (2.1%) isolate was classified as quintuple haplotype (double *Pfdhfr* with triple *Pfdhps* [CICNI-SGEGA]). In 23 (47.9%) isolates, quadruple haplotype (triple *Pfdhfr* with single *Pfdhps* [CIRNI-SGKAA]) was identified. Triple haplotypes (double *Pfdhfr* with single *Pfdhps* [CICNI-SGKAA and CNRNI-SGKAA]) were detected in 17 (35.4%) and 6 (12.5%) isolates, respectively. Finally, one (2.1%) isolate was noted to be double haplotype (single *Pfdhfr* with single *Pfdhps* [CNCNI-SGKAA]). Types of *Pfdhfr* and *Pfdhps* allele combinations are summarized in Table 3.

**Table 3:** Distribution of *Pfdhfr* and *Pfdhps* allele combinations among 48 *Plasmodium falciparum* isolates

Haplotype	*Pfdhfr*-*Pfdhps* alleles^a^	Number of the isolates, n (%)
Quintuple	CICNI-SGEGA	1/48 (2.1)
Quadruple	CIRNI-SGKAA	23/48 (47.9)
Triple	CICNI-SGKAA	17/48 (35.4)
Triple	CNRNI-SGKAA	6/48 (12.5)
Double	CNCNI-SGKAA	1/48 (2.1)

^a^*Pfdhfr*-*Pfdhps* allele combinations: quintuple (*Pfdhfr* 51I/108N + *Pfdfps* 437G/540E/581G); quadruple (*Pfdhfr* 51I/59R/108N + *Pfdfps* 437G); triple (*Pfdhfr* 51I/108N + *Pfdfps* 437G); triple (*Pfdhfr* 59R/108N + *Pfdfps* 437G); and double (*Pfdhfr* 108N + *Pfdfps* 437G) haplotype

## Discussion

In the regions where malaria is endemic, 25% of pregnant women are estimated to be infected with *Plasmodium* parasites [14]. Therefore, maintenance of IPTp-SP in those regions is crucial for protecting both pregnant women and their infants against the adverse outcomes of malaria [3]. Considering the high prevalence of malaria infection in the DRC [1], pregnant women also constitute a risk group for the disease.

Despite the effect of IPTp-SP on birth weight was evaluated previously in the DRC [2], there is a lack of research addressing the molecular basis of SP resistance. Thus, the present study was carried out to identify the *Pfdhfr* and *Pfdhps* mutations in *P. falciparum* strains isolated from 48 individuals that received two doses of SP during pregnancy.

In the study, the highest percentage (100.0%) of polymorphism in *Pfdhfr* gene was noted to be S108N, which was followed by N51I (85.4%) and C59R (60.4%) (Table 1). On the contrary, I164L mutation was not detected, and this finding is compatible with those reported elsewhere [12, 13]. The prevalence of S108N was consistent with that of a previous report (99.1%) from the DRC, however N51I and C59R rates found here differed from that study (97.9 and 80.7%, respectively) [13]. On the contrary, the rate of C59R allele was consistent with that of another study (66%) from the DRC [12]. Furthermore, findings of the present study support the hypothesis that pyrimethamine resistance is initiated by the codon 108 mutation, and improved by the additional polymorphisms at the other *Pfdhfr* codons [7].

In the present study, 47.9% of the isolates were found to harbour the triple *Pfdhfr* mutation (CIRNI haplotype) (Table 2). This is lower than previously reported rates of 57.8–78.2% [11], however similar to the findings (46.2%) of another study [10], which were both conducted in the DRC. The prevalence of CICNI (37.5%) and CNCNI (2.1%) was comparable with those reported elsewhere; however the rate of CNRNI (12.5%) in this study was found to be relatively higher [10]. In contrast with other studies where the rates of wild-type *Pfdhfr* were reported to be 1.0–7.6% [10–12], the CNCSI haplotype was not detected in the present study.

The *Pfdhps* A437G mutation was found in all (100.0%) *P. falciparum* isolates sequenced (Table 1). The prevalence of this polymorphism was reported to be 93.1% [13] and 72% [12] by former studies in the DRC. In contrast to the A437G allele, K540E in the present study was less prevalent (2.1%) than previous findings (9.5%) [13]. An increased rate of K540E polymorphism (67%) was detected by another study in Rutshuru, the DRC [12], however this was attributed to the close proximity of the study area to Eastern Africa where SP resistance is high [15]. Therefore, the result presented here supports the previous studies that documented the rate of K540E to be less than 50% in the DRC [15]. On the contrary, the rate of A581G mutation in the present study (2.1%) is different from the previous data, where it was reported to be higher than 10% in the DRC [15]. Also, no polymorphism was detected at the codon 613, which is consistent with earlier reports [12]. Taken together, the results presented here are in parallel with previous data which suggest that sulfadoxine resistance may be initiated by the mutations at codons 436 or 437, and augmented by the additional polymorphisms at the other *Pfdhps* codons [7].

In this study, SGKAA carrying the single A437G mutant allele was the most prevalent (97.9%) *Pfdhps* haplotype (Table 2). This result is higher than the recently documented percentage (76%) in the DRC [5]. On the contrary, the rate of SGEGA haplotype (437G/540E/581G) was low (2.1%) in the present study. A similar percentage (3.3%) was also noted by another study in the DRC [16]. The present result is consistent with the studies where double- and triple-mutant *Pfdhps* haplotypes were reported to be less common in Central Africa [5]. Recently, a higher percentage (8%) of SGEGA has been detected in Kinshasa (located in the west of the DRC), but this was lower compared to the eastern countries, Tanzania (15%) and Uganda (13%) [5]. Of note, a study showed that SGEGA haplotypes from the DRC and the eastern countries (Malawi and Tanzania) separated into distinct lineages. These haplotypes lead to high levels of sulfadoxine resistance, thus compromising the efficacy of SP both in pregnant women and the infants [17]. Moreover, even the haplotypes in the DRC were found to be genetically and geographically clustered. Single-mutant haplotypes were shown to be more prevalent in the west, while the double- and triple-mutant haplotypes predominated in the east of the DRC [16]. Considering that the study setting, Bandundu city, is situated in the west of the DRC, this can explain the reason of low rate of SGEGA in contrast to the high number of *Pfdhps* single-mutant haplotypes found in the present survey. On the other hand, unlike the former studies which reported the prevalence of wild-type *Pfdhps* as high as 75.6% in the DRC [11], no sensitive strain (SAKAA) was found in the current study.

The combination of *Pfdhfr*–*Pfdhps* mutant alleles generated five different haplotypes in this study. CIRNI-SGKAA was found to be the most prevalent haplotype (47.9%). This quadruple mutation was shown to occur at rates greater than 50% in African countries including the DRC. Also, this quadruple haplotype was associated with treatment failure [15].

The quintuple mutation (51I/59R/108N + 437G/540E) was previously associated with SP treatment failure and detected at rates of 27.1 and 43% in the studies from the DRC [10, 12]. Unlike those studies, here *Pfdhfr* 51I/108N allele was detected in combination with *Pfdhps* 437G/540E/581G in one (2.1%) isolate, which generated another type of quintuple mutant haplotype (CICNI-SGEGA). This haplotype was also documented by a previous study from Tanzania [9]. Owing to the association between K540E and A581G mutations and worsened SP resistance in Eastern Africa [5], the mutant strains harbouring these alleles in the DRC should be carefully considered.

The triple mutant haplotypes (CICNI-SGKAA and CNRNI-SGKAA) were identified in 35.4 and 12.5% of the isolates, respectively. A study from India found the ANRNI-SGKAA allele combination (A: alanine at codon 16 of *Pfdhfr*) in 0.92% of their isolates, and related this haplotype with low-level SP resistance [7]. Lastly, the mutant allele combination (CNCNI-SGKAA) was detected at low levels (2.1%) in the present study. This result is lower than that reported for double mutant haplotype (16.7%) in the DRC [10]. Similar to the single mutant allele in *Pfdhfr* or *Pfdhps*, the double mutant haplotype seems to occur at the initial stage of SP resistance [7].

In the present study, no *Pfdhfr*-*Pfdhps* sextuple mutant was detected, which is a promising result. Previously, sextuple haplotypes were associated with reduced birth weights [9]. Furthermore, newborns of women with the sextuple mutant haplotypes were found to have lower birth weights compared to those born to mothers with less mutated parasite infections [9]. Due to the lack of birth weight data in the study, no assessment was done between the mutation levels and the birth outcomes. Another limitation of this study is the relatively low number of participants. Yet, the mutations in both *Pfdhfr* and *Pfdhps* genes were confirmed by DNA sequencing in all 48 (100.0%) *P. falciparum* isolates. The study results also indicate that the SGEGA haplotype is present in the DRC. Considering that the SGEGA haplotypes are associated with decreased IPTp-SP outcomes [17], these mutations should be carefully monitored in the DRC.

## Conclusions

In the present study, the mutant *Pfdhfr* and *Pfdhps* haplotypes were identified in all of the *P. falciparum* strains isolated from 48 women at the time of delivery. The quadruple haplotype (CIRNI-SGKAA) which is common in Western Africa was also predominant in this study. The rate of quintuple haplotype (CICNI-SGEGA) was found to be very low. Although K540E and A581G alleles are more common in Eastern Africa, a distinct lineage of SGEGA is also present in the DRC. Therefore, despite the low levels found in this study, the triple *Pfdhps* mutation should be given particular consideration and SP resistance should be routinely monitored in the DRC. To the authors’ knowledge, this is the first report from the DRC that addressed the molecular determinants of SP resistance in pregnancy. This study would provide a basis for future research to evaluate the correlation of mutation levels and the clinical outcomes of IPTp-SP in the DRC.
